# Complete Genome Sequence of *Helicobacter pylori* Strain 29CaP Isolated from a Mexican Patient with Gastric Cancer

**DOI:** 10.1128/genomeA.01512-15

**Published:** 2016-01-14

**Authors:** Eduardo Mucito-Varela, Gonzalo Castillo-Rojas, Miguel A. Cevallos, Luis Lozano, Enrique Merino, Gamaliel López-Leal, Yolanda López-Vidal

**Affiliations:** a Departamento de Microbiología y Parasitología, Facultad de Medicina, Programa de Inmunología Molecular Microbiana, Universidad Nacional Autónoma de México (UNAM), Ciudad de México, Distrito Federal, Mexico; b Centro de Ciencias Genómicas, Programa de Genómica Evolutiva, Universidad Nacional Autónoma de México (UNAM), Cuernavaca, Morelos, Mexico; c Instituto de Biotecnología, Universidad Nacional Autónoma de México (UNAM), Cuernavaca, Morelos, Mexico; d Facultad de Medicina, División de Investigación, Universidad Nacional Autónoma de México (UNAM), Ciudad de México, Distrito Federal, Mexico

## Abstract

*Helicobacter pylori* infection is a risk factor for the development of gastric cancer and other gastroduodenal diseases. We report here the complete genome sequence of *H. pylori* strain 29CaP, isolated from a Mexican patient with gastric cancer. The genomic data analysis revealed a *cag*-negative *H. pylori* strain that contains a prophage sequence.

## GENOME ANNOUNCEMENT

*Helicobacter pylori*, a Gram-negative bacterium, infects half of the population worldwide (1, 2) and contributes to the development of diverse gastric diseases. Despite *H. pylori* infection being necessary but not sufficient for the development of diseases, infection with this bacterium is highly associated with gastric pathologies, including gastric cancer (3). Gastric cancer is a major cause of cancer death worldwide (4) and the third highest cause of cancer related to *H. pylori* infection in Mexico (5). However, circulating *H. pylori* strains in the Mexican population are poorly studied. Hence, there is a need for studies aimed at understanding *H. pylori* strains among the Mexican population.

We announce here the complete sequence genome of *H. pylori* strain 29CaP, isolated from a biopsy sample from a Mexican patient with advanced gastric adenocarcinoma. Total DNA sequencing was performed using three different technologies: PacBio RS, Illumina MiSeq 2000, and 454 Titanium. We obtained an average sequencing coverage of 135×. The quality control reads and *de novo* assembly were performed using the FastQC (http://www.bioinformatics.babraham.ac.uk/projects/fastqc/), FASTX-Toolkit (http://hannonlab.cshl.edu/fastx_toolkit/), and SMRT (version 2.3)-MIRA assembler (version 3.4) tools, respectively. We closed the genome with a total assembly size of 1.6 Mb, with a G+C content of 38%.

Finally, we performed the genome annotation in GenBank using the NCBI Prokaryotic Genome Annotation Pipeline (http://www.ncbi.nlm.nih.gov/genome/annotation_prok/). Overall, the annotation revealed a total of 1,615 genes, 117 pseudogenes, 36 tRNAs, 7 rRNAs, and 1 noncoding RNA (ncRNA). Additionally, sequence analysis of this strain showed the absence of the *cag* pathogenicity island, an s2i2m2 VacA genotype, and the presence of a complete prophage sequence of 31.7 kb. This genome and other sequenced genomes will be used for computational studies.

### Nucleotide sequence accession number.

This whole-genome shotgun project has been deposited in GenBank under the accession number CP012907.
